# Enhanced bioavailability of Quercetin-loaded niosomal in situ gel for the management of Parkinson’s disease

**DOI:** 10.3389/fphar.2024.1519649

**Published:** 2025-01-27

**Authors:** Abhishek A. Revankar, Archana S. Patil, Reshma Karishetti, Krutuja R. Chougule, Priyanka Patil, Abhijit Salokhe

**Affiliations:** ^1^ Department of Pharmaceutics, KLE College of Pharmacy, KLE Academy of Higher Education and Research, Belagavi, Karnataka, India; ^2^ Department of pathology, Jawaharlal Nehru Medical College, KLE Academy of Higher Education and Research, Belagavi, Karnataka, India; ^3^ Department of Pharmacology, KLE College of Pharmacy, KLE Academy of Higher Education and Research, Belagavi, Karnataka, India

**Keywords:** biochemical assay, intranasal, niosomes, Parkinson disease, quercetin

## Abstract

**Background:**

Parkinson’s disease (PD) is the second most prevalent neurological disorder, characterized by motor symptoms such as tremor and rigidity due to the degeneration of dopaminergic neurons in the substantia nigra. This study investigates the formulation of quercetin, a natural bioflavonoid with potent antioxidant and anti-inflammatory properties, as niosomes for intranasal delivery to enhance its bioavailability and therapeutic potential for PD.

**Methods:**

The niosomal formulation was optimized for critical parameters including particle size, entrapment efficiency, and zeta potential. Male Wistar rats were utilized to assess the effects of quercetin-loaded niosomes on motor function, dopaminergic neuron protection, and oxidative stress alleviation.

**Results:**

The optimized niosomal formulation exhibited a particle size of 195 nm, a polydispersity index (PDI) of 0.29, a zeta potential (ZP) of −30.63 mV, and an entrapment efficiency (EE) of 82.77%. *In vivo* evaluations conducted using the haloperidol-induced PD model revealed significant enhancements in behavioural, biochemical, and histopathological outcomes when compared to both disease controls and the standard treatment group. Additionally, short-term stability tests confirmed the robustness of the formulation.

**Conclusion:**

The findings suggest that the quercetin-loaded niosomal formulation offers improved drug delivery and efficacy, indicating its potential as a superior treatment option for PD compared to conventional dosage forms. This approach may pave the way for enhanced therapeutic strategies targeting the neurodegenerative processes underlying Parkinson’s disease.

## 1 Introduction

Parkinson’s disease (PD) ranks as the second most common neurodegenerative disorder, primarily manifesting through hallmark motor symptoms such as tremor, bradykinesia, postural instability, and rigidity, with non-motor symptoms including sleep disturbances, olfactory deficits, cognitive impairments, and depression often predating the motor manifestations (Madiha et al., 2021). The neuropathological underpinnings of PD involve progressive degeneration of dopaminergic neurons within the substantia nigra pars compacta, culminating in a marked dopamine (DA) deficit in the striatum. This dopaminergic depletion, coupled with alterations in other monoamines (e.g., serotonin, norepinephrine), drives the symptomatic profile of PD, both motor and non-motor (Madiha et al., 2021). Although PD’s etiology remains largely enigmatic, evidence implicates genetic mutations, environmental toxins, oxidative stress, mitochondrial impairment, and neuroinflammation in its pathogenesis (Madiha et al., 2021). Current therapeutic strategies are primarily symptomatic, leveraging dopaminergic agents like levodopa, whose efficacy, however, wanes over time and is often accompanied by adverse effects (Jiang et al., 2023; Mitsuboshi et al., 2022).

Quercetin, a polyphenolic bioflavonoid widely distributed in fruits and vegetables, exhibits potent antioxidant and anti-inflammatory properties, offering a promising neuroprotective potential for PD management (Wrobel-Biedrawa et al., 2022; Grewal et al., 2021). It is known to neutralize reactive oxygen species (ROS), bolster endogenous antioxidant defenses, and modulate inflammatory cascades, presenting an ideal candidate for neuroprotection (Wrobel-Biedrawa et al., 2022). Additionally, quercetin has shown efficacy in curbing α-synuclein aggregation, a pathological hallmark associated with neurodegeneration in PD (Grewal et al., 2021). However, quercetin is categorized in a Biopharmaceutical Classification System (BCS) as Class IV (Sirvi et al., 2022) compound, indicating poor solubility and permeability. These limitations significantly impede its therapeutic potential, necessitating innovative formulation strategies to enhance its bioavailability. Niosomal encapsulation offers a promising approach, as it can improve Quercetin’s solubility and permeability, thus optimizing its delivery and bioefficacy in PD models (Madiha et al., 2021; Nirmal et al., 2010). Preclinical studies corroborate quercetin’s ability to ameliorate motor function, preserve dopaminergic neurons, and mitigate oxidative stress in PD animal models (Grewal et al., 2021). However, transitioning these findings into clinical settings demands further research, particularly in optimizing delivery methods to improve quercetin‘s therapeutic viability (Nirmal et al., 2010).

Niosomes, composed of a nonionic surfactant shell and an aqueous core, exhibit enhanced chemical and physical stability over liposomes, making them suitable for diverse drug delivery applications (Rajoria and Gupta, 2012). Given their versatility, niosomes are particularly advantageous for nasal delivery, boasting compatibility with both hydrophilic and lipophilic drugs, high biocompatibility, low toxicity, and enhanced permeability across biological membranes. These attributes render niosomes an appealing carrier for quercetin, enhancing its stability and membrane penetration capabilities, particularly when incorporated into an intranasal delivery system (Elkomy et al., 2023). The intranasal delivery of quercetin-loaded niosomal *in situ* gel offers direct central nervous system (CNS) access via the olfactory and trigeminal pathways, minimizing systemic side effects (Gastrointestinal Irritation, Hepatotoxicity, Renal Stress) while enhancing bioavailability. Moreover, an *in situ* mucoadhesive gel can facilitate prolonged nasal residence time, thereby augmenting drug absorption and therapeutic outcomes (Hu et al., 2022; Teaima et al., 2022).

Previous attempts to utilize quercetin in Parkinson’s disease treatment have involved various formulations; (Acıkara et al., 2022; Tamtaji et al., 2020); however, these have often been limited by low oral bioavailability and a lack of targeted brain delivery. In this study, we have developed an intranasal *in situ* gel formulation of quercetin that is designed to bypass these limitations, offering direct brain targeting and enhanced bioavailability. This approach leverages the intranasal route to facilitate efficient drug delivery to the central nervous system, potentially improving therapeutic outcomes for Parkinson’s patients.

## 2 Materials and methods

A pure sample of Quercetin was sourced from Kemphasol, Mumbai, India with haloperidol provided as a gift sample by Vamsi Labs Ltd., and L-dopa procured from Otto Chemicals, India. Cholesterol was obtained from Loba Chemie, while dialysis membranes (molecular weight cutoff range 12,000–14,000 g/mole) and dialysis tubing were sourced from Hi-Media Laboratories, Mumbai, India. Poloxamer 407 (P407) and benzalkonium chloride were procured from Sigma Aldrich Pvt. Ltd., Mumbai, India.

### 2.1 Preparation of niosomes

Niosomes were formulated via the ethanol injection method. A fixed amount of Quercetin, along with varying ratios of Span (Sp) and cholesterol (CH) as shown in Table 1, was dissolved in 10 mL of ethanol. The organic solution was subsequently injected into 10 mL of Type 1 Millipore water using a 22-gauge needle attached to an injection pump, maintaining a constant flow rate of 20 drops per minute. The resultant mixture was stirred magnetically at 65°C on a hotplate magnetic stirrer (MS-H550-Pro, Medfuture Biotech Co., Jinan, China) for 1 hour, ensuring complete ethanol evaporation, leading to niosome formation. To achieve smaller particle sizes, the dispersion underwent ultrasonic probe sonication for 5 min at a 30% pulse rate (Glascock et al., 2011).

**TABLE 1:** Formulation design parameters for the optimization of Quercetin-loaded niosomes using box-behnken design.

Independent variables			
Span 60 (mg)	30	45	60
Cholesterol (mg)	20	30	10
Sonication time (Mins)	5	10	15
Dependent variables			
Particle size	Minimum		
%Entrapment efficiency	Maximum		
Zeta potential	In range		

### 2.2 Experimental design

The Design Expert^®^ software (version 13, Stat-Ease Inc., Minneapolis, MN, United States) was employed to create a three-factor, three-level (3³) Box-Behnken Design (BBD) with five center points, resulting in 17 experimental runs as depicted in Table 1. This design, favored for its efficiency in generating higher-order response surfaces with fewer runs, was used to optimize Quercetin -loaded niosomes with Span 60 (X1), Cholesterol (X2), and Sonication time (X3) as independent variables. The response variables included particle size (Y1), entrapment efficiency (Y2), and zeta potential (Y3) (Eid et al., 2022). Optimization utilized constraints aimed at maximizing nasal membrane permeation, minimizing particle size, and enhancing entrapment efficiency to increase payload capacity. The batch with the highest desirability was selected as the optimized formulation. Factor levels were selected by preliminary studies to maximize entrapment efficiency in niosomal dispersions (Elkomy et al., 2022; Elkomy et al., 2017).

### 2.3 Characterization of quercetin-loaded niosomal dispersion

#### 2.3.1 Determination of PS, ZP, and PDI

Particle size, PDI and Zeta potential values of Quercetin loaded niosomes were measured physicochemically using the dynamic light scattering technique in accordance with standard protocols. The Nano ZSU3100 (Malvern Instruments, United Kingdom) was the instrument used for this assessment. The niosomes suspensions were combined with Millipore water type 1 (1:10), The assessment was carried out at room temperature, which was 25°C ± 0.5°C (Teaima et al., 2022). The triplicate mean ± SD was used to report the results.

#### 2.3.2 Determination of entrapment efficiency

The EE% of QUE-loaded niosomes was determined indirectly by calculating the difference between the total amount of quercetin incorporated into formulation and the amount that remained in the supernatant after separating the prepared vesicles by centrifugation at 15,000 rpm for 1 h at 4 °C using cooling centrifuge. The concentration of free quercetin was measured spectrophotometrically by measuring the ultraviolet (UV) absorbance, at λ max 256 nm (Shilakari Asthana et al., 2016). Drug EE% was calculated according to the following equation.

$$EE=\frac{\text{Total}\;\text{amount}\;\text{of}\;\text{drug}\;\text{added}\;–\text{Amount}\;\text{of}\;\text{drug}\;\text{in}\;\text{supernant}}{\text{Total}\;\text{amount}\;\text{amount}\;\text{of}\;\text{drug}\;\text{added}}\times 100$$

#### 2.3.3 Morphological analysis by transmission electron microscopy (TEM)

To perform Transmission Electron Microscopy (TEM) analysis using the TECNAI G2 SPIRIT BIOTWIN, dilute 0.5 μL of the sample with 4.0 mL of distilled water. Pipette 0.2 μL of the diluted sample onto a 200-mesh copper-carbon grid. Stain the grid with 2% Phosphotungstic Acid (PTA) and let it dry at room temperature. Place the dried grid into the TEM sample holder. Use the Tecnai Imaging and Analysis software to capture images and perform measurements.

#### 2.3.4 *In vitro* release study

*In vitro* release of niosomes suspension was carried out by dialysis bag method. A dialysis sac was washed and soaked in distilled water. Vesicle suspension was pipetted into a bag made up of tubing and sealed followed by placing the dialysis bag into a beaker containing 90 mL of PBS pH. The vessel was placed over magnetic stirrer (50 rpm) and the temperature was maintained at 37^∘^C ± 0.5^∘^C. Samples were withdrawn at predetermined time intervals and immediately replaced with the fresh medium to maintain the sink condition throughout experiment. Samples were diluted and analyzed for drug content by using UV/visible spectrophotometer at 256 nm (Schmolka, 2014).

#### 2.3.5 Stability studies of optimized formulation

To determine the stability of the optimized niosomes, short term stability studies were conducted in compliance with ICH GCP guidelines. The prepared formulation was stored in glass vials within a humidity-controlled oven maintained at a temperature of 25 ± 2°C and relative humidity of 65% ± 5%. Additionally, it was refrigerated at 4 ± 2°C with relative humidity of 65% ± 5%. At regular intervals 0, 30 and 90 days, a sample was taken for analysis.

#### 2.3.6 Formulation of *in situ* nasal gel of quercetin loaded niosomes

To enhance the formulation, optimized niosomes were incorporated into an *in situ* gel to extend nasal residence time, improve physical stability, and increase patient acceptability. The thermosensitive *in situ* gel was prepared using a modified cold method, where the gelling agent poloxamer 407 was dispersed within the optimized niosomal dispersion. The formulation was stored at 4°C for further study. The *in situ* gel’s characteristics were assessed, including flowability, pH, viscosity, transparency, gelation temperature, gelation time, and *in vitro* drug diffusion properties (Galgatte et al., 2014).

### 2.4 Evaluation of niosomal *in situ* gel

#### 2.4.1 Determination of pH

The pH of niosomal *in situ* gel was determined by digital pH meter. 1 gm of gel was placed in distilled water (25 mL) and the pH was measured using pH meter.

#### 2.4.2 Determination of flowability of niosomal *in situ* gel

Test tube inversion method was used to determine the Transition of sol to gel of the niosomal *in situ* gel (NIG). NIG (2 mL) was transferred to a test tube, covered with paraffin and then inverted. It was maintained at 32°C in the water bath at a steady temperature and the flowability of the sample was analysed (Afreen et al., 2022).

#### 2.4.3 Determination of viscosity

The viscosity of the prepared gel formulations was assessed using a Brookfield DV-II Pro Plus viscometer equipped with a T-bar spindle. Viscosity measurements were performed across various temperatures and shear rates. To examine temperature-dependent behavior, the formulation was subjected to a constant shear rate within a temperature range of 25°C–40°C. The viscosity of the sol state was measured at room temperature (25°C) using spindle L2, while gel state measurements were conducted at nasal temperature (34°C) using spindle L3, due to the observed increase in viscosity. Viscosity values were calculated as the mean of three replicates to ensure accuracy (Afreen et al., 2022).

#### 2.4.4 *In vitro* drug diffusion study

The Franz diffusion system was used to measure the drug release from a gel using a cellophane dialysis membrane. Before beginning the experiment, cellophane membrane fragments were placed and soaked in a receptor medium of phosphate buffer at pH 6.4 for 2 hours. The cellophane membranes, with effective permeation areas of 2 × 2 cm^2^, were then stationed onto the Franz diffusion cells. The donor compartment was filled with 2.5 mg (200 μL) of quercetin-loaded niosomal gel, while the receptor compartment was filled with 12 mL of PBS at pH 6.4. The experiment was carried out at a temperature of 34°C ± 1°C with constant stirring. An aliquot of 0.5 mL was taken from the receptor compartment at different time intervals: 0.5, 1, 2, 4, 6, 8, 12, and 24 h. The samples were appropriately diluted and analyzed at 256 nm using UV-Visible spectroscopy. The *in vitro* release experiments were analyzed kinetically using different mathematical models, including Zero order, First order, Korsmeyer–Peppas, Higuchi and Hixson Crowell, to determine the goodness of fit in terms of *R* ^2^ values (Saleem et al., 2021).

#### 2.4.5 *In vivo* pharmacodynamic study design

The study protocol was reviewed and approved by the Institutional Animal Ethical Committee of KLE COP, Belagavi (accession number: 221/Po/Re/S/2000/CPCSEA) Male Wistar rats weighing 150–200 g were used in the experiment and were categorized into 4 groups having 6 animals in each group. The rats were divided into four groups, each consisting of six rats.

1. Normal group: Received food and water only.
2. Control group: Administered Haloperidol at 1.25 mg/kg via intraperitoneal injection (i.p.) for 21 days.
3. Standard group: Given Haloperidol at 1.25 mg/kg (i.p.) in combination with L-dopa at 10 mg/kg for 21 days.
4. Niosomal *In situ* gel group: Treated with Haloperidol at 1.25 mg/kg (i.p.) for 21 days along with Quercetin (niosomes *in situ* gel) intranasally (0.5 mL) for 21 days.

**FIGURE 1:**
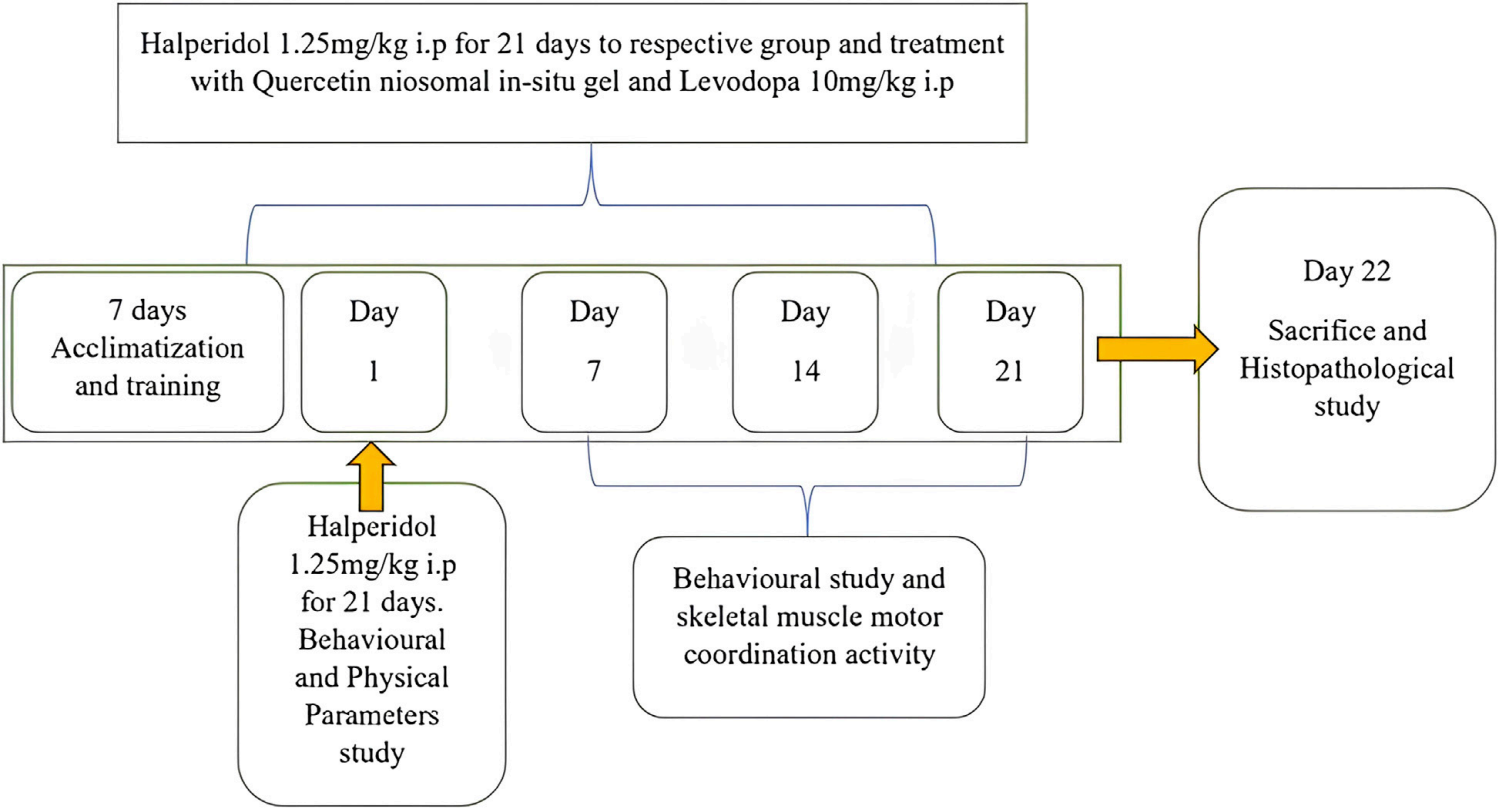
Schematic description of the experimental plan.

Quercetin niosomal *in situ* gel was administered intranasally at a dose of 10 mg/kg. L-dopa (standard) was administered intraperitoneally (I.P.) at a dose of 10 mg/kg. The animals were trained using the rotarod, actophotometer, balance beam, and catalepsy tests for acquisition. After administering the test agents, changes in latency were examined on the 7th (first dose), 14th, and 21st days. Twenty-five days after the last dose, the normal, control, standard, and treatment groups were euthanized, and their brains were removed for further histopathological and biochemical estimations, including reduced GSH, MDA, and SOD (Shrivastava et al., 2013). Experimental plan is displayed in Figure 1.

#### 2.4.6 *In vivo* screening models for Parkinson disease

##### 2.4.6.1 Behavioral testing

Behavioral tests like Rotarod, Balance Beam, and Actophotometer are used in Parkinson’s disease research to measure motor coordination, balance, and activity levels in animal models, providing insights into disease progression and evaluating potential therapies (Hira et al., 2020).

###### 2.4.6.1.1 Rotarod analysis

The rotarod device was used to assess the grip strength. In order to evaluate “minimal cognitive deficits” like balance and motor control, this tool is widely used. Before starting therapy, each rat underwent a training session to become acclimated to the apparatus. The animal was fastened to a rotating rod with a 7 cm diameter and 25 revolutions per minute. After being given Quercetin and Haloperidol, each rat was put on a rotating rod with a 180-second cutoff time. There was an expression for the latency time before the data started to fall (Hira et al., 2020).

###### 2.4.6.1.2 Balance beam analysis

A balance beam device was used to evaluate dyskinetic gait abnormalities. The apparatus consists of a start and finish box-equipped 1-meter-long and 1-cm-wide runway. Foam padding was positioned 50–60 cm above the ground beneath the beam to safeguard the animals. Before the test, the animals were trained, and at the conclusion of the walk, they were given food pellets as a reward. Every 7 days, tests were carried out, and the amount of time it took to get to the end box was measured and recorded in seconds (Kondawar et al., 2011).

###### 2.4.6.1.3 Actophotometer analysis

Animals are forced to move from one location to another by a physical process known as locomotion. Using an actophotometer, this skill can be easily evaluated (activity cage). It's a device made up of photoelectric cells. When an animal in a cage interrupts the light beam falling on the photocell, a count of the animal’s movements is displayed on the screen. Every animal was trained for the test 1 day prior to it, and it was conducted every 7 days. The results were expressed in counts/5 min and the cutoff time was 5 min (Ranpariya et al., 2011).

##### 2.4.6.2 Preparation tissue homogenate

Brains were separated and thoroughly cleaned after animals were sacrificed. One gram of brain was homogenized with Tris-buffer (pH 7.4) using a homogenizer. After that, it was centrifuged for 15 min at 7,000 rpm in cold centrifugation. For additional estimation, the supernatant was obtained and kept in sterile Eppendorf tubes. The supernatant obtained was used to estimate Malondialdehyde (MDA) (Hira et al., 2019), superoxide dismutase (SOD) (Ellman, 1959), glutathione (GSH) (Bhangale and Acharya, 2016; Abdelbary and Abd-Elsalam, 2019).

##### 2.4.6.3 Statistical analysis

The data for the Rotarod, Actophotometer, and Balance Beam tests, as well as the biochemical assays (GSH, MDA, SOD), are presented as mean ± SEM (n = 6). All data were analyzed using appropriate statistical methods. Specifically, two-way ANOVA with Tukey’s multiple comparisons test was applied for the behavioral assessments (Rotarod, Actophotometer, and Balance Beam tests), and one-way ANOVA with Dunnett’s multiple comparisons test was used for the biochemical assays (GSH, MDA, SOD). The *p*-values for all the behavioral results were less than 0.001, indicating highly significant differences between the disease group and the treatment groups.

## 3 Results

### 3.1 Formulation of quercetin-loaded niosome using a Box-Behnken experimental design

Quercetin-loaded niosomes were successfully prepared utilizing a modified ethanol injection technique. A total of 17 formulations were developed using Span 60, cholesterol, and varying sonication times. The formulations were optimized through a Box-Behnken experimental design, as detailed in Table 2.

**TABLE 2:** Experimental design for formulations based on the box-behnken design (3^3^).

Run	Factor 1 A: Span 60 in mg	Factor 2 B: Cholesterol in mg	Factor 3 C:Sonication time	Response 1 PS (nm)	Response 2 EE (%)	Response 3 ZP (mV)
1	30	20	10	155	68	−22
2	30	30	15	165	75	−26.8
3	30	40	10	220	84	−35
4	30	30	5	220	84.2	−22
5	45	30	10	170	76	−23
6	45	30	10	220	78	−26.49
7	45	30	10	195	81	−24.7
8	45	30	10	220	80	−29.4
9	45	40	15	210	81.7	−28.9
10	45	20	15	142	60	−34
11	45	20	5	170	68.9	−19.4
12	45	30	10	201	83	−18
13	45	40	5	276	81.8	−29.4
14	60	30	5	215	70.36	−33
15	60	40	10	180	83	−39
16	60	30	15	200	70.4	−25.8
17	60	20	10	128	71	−17.8

### 3.2 Response surface analysis

#### 3.2.1 Response surface plot showing the effect of concentration of span 60 (A), cholestrol (B) and sonication time (C) on particle size (PS)

For effective brain targeting, it is critical to maintain the particle size (PS) of niosomes within the range of 100–300 nm. The PS values produced for niosomes were analyzed using a polynomial quadratic model, demonstrating adequate precision (10.52) and a reasonable difference between the predicted *R* ^2^ (0.5745) and the adjusted *R* ^2^ (0.7708), with a discrepancy of less than 0.2. Observed particle sizes ranged from 128 nm to 270 nm. The final equation for PS analysis, expressed in terms of coded factors, highlighted the influences of Span 60 (A), Cholesterol (B), and Sonication time (C).

Cholesterol significantly influences niosomal size by increasing vesicle rigidity and stability. As the cholesterol concentration rises, it competes with the drug for space within the bilayer, leading to a larger hydrodynamic diameter and increased particle size due to strengthened bilayer structure (Shah et al., 2020). Additionally, cholesterol’s mechanism includes increasing chain order in liquid-state bilayers while reducing it in gel-state bilayers, transforming the gel phase gradually into a liquid-ordered state. This shift favours encapsulation efficiency for hydrophilic drugs and decreases the release rate of the entrapped drug by enhancing membrane stability (Uchegbu and Vyas, 1998).

**FIGURE 2:**
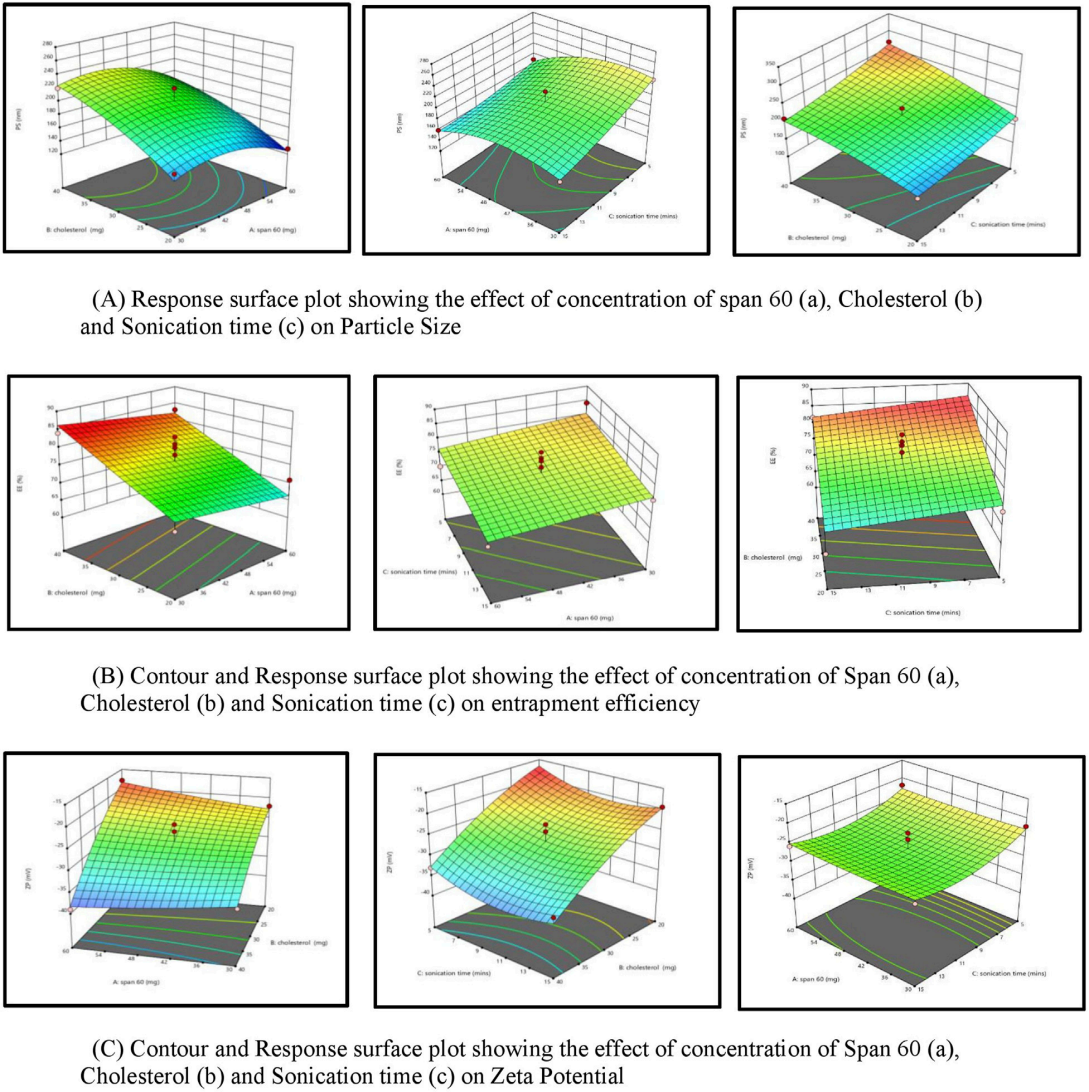
**(A)** Response surface plot depicting the effect of span 60 concentration (a), cholesterol (b), and sonication time (c) on particle size. **(B)** Contour and response surface plot showing the influence of span 60 concentration (a), cholesterol (b), and sonication time (c) on entrapment efficiency. **(C)** Contour and response surface plot depicting the effect of span 60 concentration (a), cholesterol (b), and sonication time (c) on zeta potential.

In contrast, increasing Span 60 concentration and sonication time reduces particle size. Span 60, with its long alkyl chain (C18), lowers surface tension, facilitating smaller vesicle formation, while also contributing to bilayer rigidity, thus stabilizing smaller, uniform particles (Essa, 2010; Owodeha-Ashaka et al., 2010). Sonication, by applying ultrasound waves, induces cavitation, breaking larger vesicles into smaller, more uniform ones (Khallaf et al., 2020). This combination of Span 60 and sonication supports the creation of optimized niosomes with suitable PS for brain delivery (Abdelkader et al., 2014; Shah et al., 2020; Nasr et al., 2020). Response surface plot showing the effect of concentration of Span 60 (A), Cholestrol (B) and Sonication time (C) on particle size (PS) is depicted in Figure 2.

$$\text{PS}=+201.20-14A+35.63B-22.88C-3.25\text{AB}+8.75\text{AC}-8\text{BC}-21.10A^{2}-9.3.5B^{2}+6.15C^{2}$$

#### 3.2.2 Response surface plot showing the effect of concentration of span 60 (A), cholesterol(B) and sonication time (C) on entrapment efficiency (EE%)

Entrapment efficiency (EE%) is a critical measure indicating the proportion of a drug successfully encapsulated within nanocarriers, ensuring more of the drug is available to exert its therapeutic effects at the target site. In this study, the EE% ranged from 60% to 84.2%. The EE% values of the generated niosomes were analyzed using a linear model with an adequate precision of 9.7. The difference between the predicted *R* ^2^ (0.4883) and the adjusted *R* ^2^ (0.6387) was less than 0.2, indicating a reasonable model fit.

An increase in cholesterol concentration positively influences encapsulation efficiency (EE%) due to Cholesterol’s significant role in enhancing drug encapsulation within the vesicle membranes. A higher percentage of Cholesterol correlates with an increased EE%, as Cholesterol modulates the mechanical strength, cohesion, and permeability of lipid bilayers in niosomes. Specifically, Cholesterol reduces the fluidity of niosomes, thereby imparting rigidity to the vesicles, which results in less permeable niosomes—a critical attribute under high-stress conditions (Moghassemi and Hadjizadeh, 2014). Conversely, an increase in Span 60, coupled with increased sonication, adversely affects entrapment efficiency. This phenomenon arises from the interaction between Cholesterol and Span 60 within the bilayer of the vesicles, primarily due to hydrogen bonding (Moghassemi and Hadjizadeh, 2014). Span 60, characterized by its long alkyl chain (C18) and low water solubility, facilitates tight packing within the niosomal bilayer (Uchegbu and Vyas, 1998). While this tight packing aids in reducing overall vesicle size, it may also restrict the volume of drug that can be encapsulated (Masjedi and Montahaei, 2021). Cholesterol effectively cements the leakage pathways within the bilayer membranes, resulting in a firmer membrane structure as the surfactants become more densely packed during the filling process (38). Response surface plot showing the effect of concentration of span 60 (A), Cholestrol (B) and Sonication time (C) on Encapsulation Effeciency (%EE) is depicted in Figure 2.

EE=+79.60−2.06A+7.83B−2.26C

#### 3.2.3 Response surface plot showing the effect of concentration of span 60 (A), cholesterol (B) and sonication time (C) on zeta potential (ZP)

The zeta potential (ZP) of the developed niosomal dispersion was analyzed to elucidate the surface charge characteristics, which are pivotal for predicting the colloidal stability of the dispersion. Elevated absolute ZP values provide adequate electrostatic repulsion to mitigate particle aggregation. The analysis revealed that quercetin-loaded niosomes exhibited a negative surface charge, with average values ranging from −39 mV to −17.8 mV. This assessment was conducted using a polynomial quadratic model, which demonstrated an adequate precision of 11.2 and a reasonable disparity between the predicted *R* ^2^ (0.7078) and the adjusted *R* ^2^ (0.6387), with the difference being less than 0.2. The inclusion of cholesterol enhances the magnitude of the negative surface charge on the niosomes. This effect arises from cholesterol’s capacity to interact with the charged head groups of surfactants, such as dicetylphosphate, thereby contributing to the negative zeta potential (Junyaprasert et al., 2008; Zolghadri et al., 2023). The type of surfactant, the presence of charged species, and storage conditions are significant formulation parameters that influence the zeta potential of niosomes (Durán-Lobato et al., 2022). An increase in the amount of Span 60 within niosomes typically correlates with a decrease in the magnitude of the zeta potential. This decrease can be attributed to the hydrophobic nature of the surfactant, which diminishes the electrostatic repulsion among the niosomes, leading to reduced stability and an increased propensity for aggregation (Shah et al., 2012). However, sonication time may not be a major determinant compared to these other parameters. Further targeted research is warranted to conclusively elucidate the impact of sonication time on zeta potential. The quadratic equation of factors affecting ZP is shown below. Response surface plot showing the effect of concentration of span 60 (A), Cholestrol (B) and Sonication time (C) on zeta potential (ZP) is depicted in Figure 2.

$$\text{ZP}=-27.08+0.1750A-7.98B-1.40C-2.05\text{AB}+0.2\text{AC}+0.1000\text{BC}+0.3150A^{2}-1.69B^{2}+2.66C^{2}$$

### 3.3 Analysis of ANOVA of calculated response

Analysis of variance of calculated Responses and summary of the results of responses of particle size, entrapment efficiency, and Zeta potential are depicted in Table 3, 4 respectively.

**TABLE 3:** Statistical analysis of model fit for particle size, entrapment efficiency, and zeta potential.

Source	Standard deviation	*R* ^2^	Adjusted *R* ^2^	Predicted *R* ^2^	Press	
Particle size						
Linear	19.88	0.7559	0.6996	0.5978	8462.51	Suggested*
2FI	21.29	0.7847	0.6555	0.3457	13769.29	
Quadratic	17.36	0.8997	0.7708	0.5745	8953.63	Suggested*
Cubic	20.73	0.9183	0.6733			
Entrapment efficiency						
Linear	4.25	0.7064	0.6387	0.4883	409.14	Suggested*
2FI	4.36	0.7627	0.6203	0.2081	633.19	
Quadratic	3.53	0.8910	0.7508	−0.2173	973.27	
Cubic	2.70	0.9635	0.8539			
Zeta potential						
Linear	2.80	0.8375	0.799	0.7186	176.31	Suggested*
2FI	2.91	0.8646	0.7834	0.5491	282.55	
Quadratic	2.52	0.9293	0.8385	0.70798	183.10	Suggested*
Cubic	3.02	0.9419	0.7677			

*Equation models suggested by the Design of Expert Software.

**TABLE 4:** Response results for particle size, entrapment efficiency, and zeta potential.

Result of ANOVA	Particle size	Entrapment efficiency	Zeta potential
Sum of square	18932.51	564.83	582.30
Degree of freedom	9	3	9
Mean square	2103.6	188.8	64.70
F-value	6.98	10.43	10.23
P-value	0.0090	0.0009	0.0059
Inference	Significant	Significant	Significant

### 3.4 Formulation optimization

**FIGURE 3:**
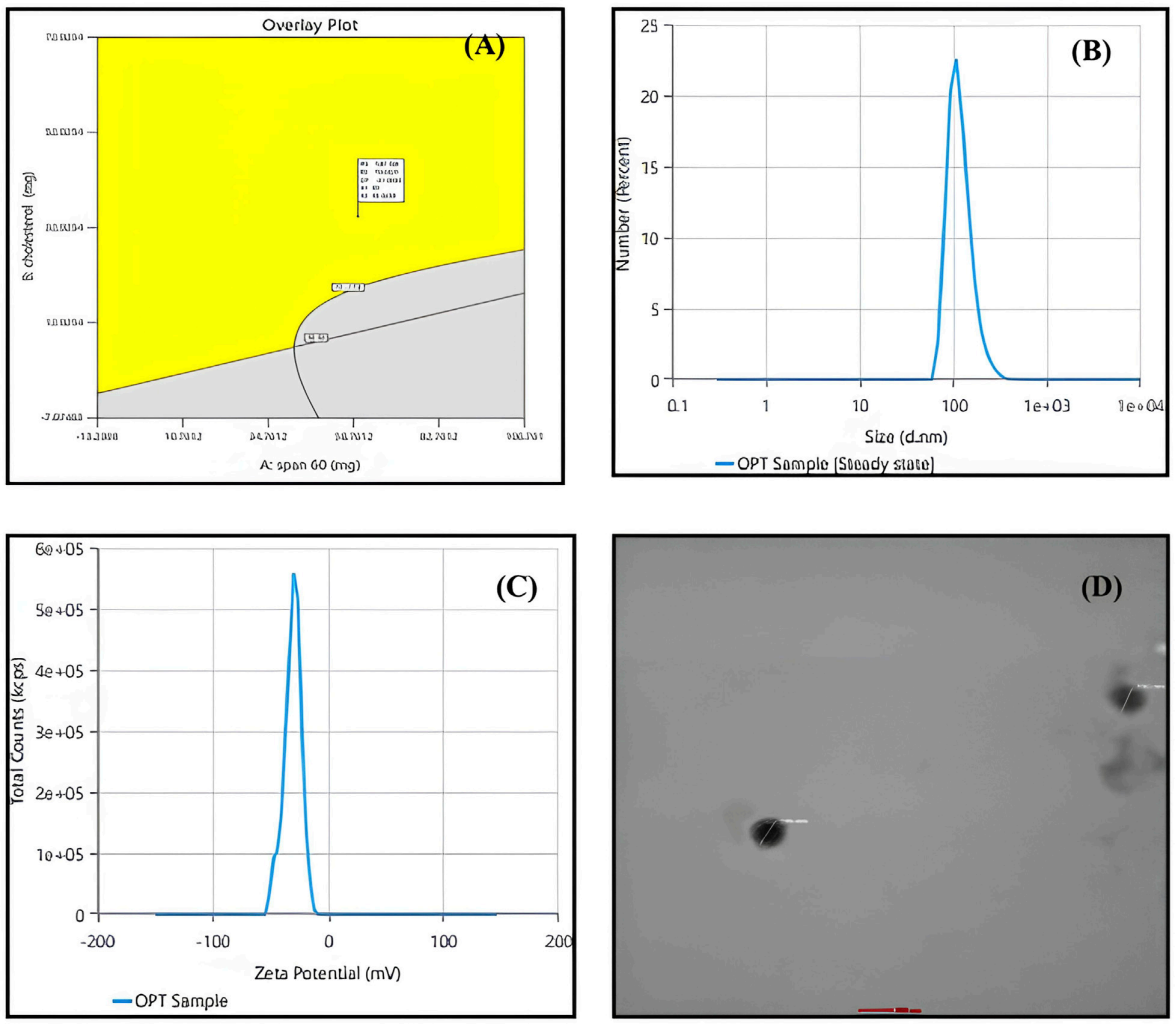
**(A)** overlay plot indicating the optimized box-behnken experimental design conditions marked within the design space. **(B)** Particle size peak report from zetasizer indicating 195.9 nm for the optimized niosomes dispersion. **(C)** Zeta potential peak report from zetasizer indicating −30.63 mV for the optimized niosomes dispersion. **(D)** transmission electron microscopy (TEM) image of optimized quercetin-loaded niosomes.

The Design expert^®^ software provided a range of suggestions that optimally satisfied the set constraints. The selected formula had a desirability value of 0.821. Table 5 displays the values of experimental, predicted value for the response variables of the optimized Quercetin loaded niosomes formula. These findings demonstrated the validity of the final models. Overlay plot showing optimized box Behnken experimental design conditions as a flag within the design space is illustrated in Figure 3. Peak report depicting particle size and zeta potential for the optimized niosomes dispersion is displayed in Figure 3.

**TABLE 5:** Validation results of the optimized formulation.

	Independent variable			Responses			Desirability
	Span 60 in mg	Cholesterol in mg	Sonication time (in mins)	VS(nm)	EE(%)	ZP(mV)	
Solution (Predicated values)	60	35.34	8.433	187.10	79.08	−31.810	0.821
Practically performed	60	35.34	8.433	195.9	82.77	−30.63	

### 3.5 Morphological analysis by transmission electron microscopy (TEM)

Niosomal morphological analysis was conducted using Transmission Electron Microscopy (TEM), as illustrated in Figure 3. The resulting images clearly demonstrate that the Quercetin-loaded niosomes exhibit a spherical morphology with a homogeneous size distribution, indicative of the stability of the prepared niosomes. The niosomes displayed a well-defined spherical shape characterized by a distinct wall structure. The stability of the multiple bilayers, along with their rigidity, is primarily governed by the hydrogen bonding interactions between the ester groups of Span 60 and the hydroxyl groups of cholesterol (Syed et al., 2021; El-Diasty et al., 2020).

### 3.6 *In Vitro* release and kinetic analysis of quercetin loaded niosomes

The *in vitro* release profile of quercetin from the optimized niosomal formulation displayed a marked biphasic pattern across the 24-hour assessment period. The release commenced with a rapid “burst” phase within the first 2 hours, during which approximately 29.56% of quercetin was discharged from the niosomes. This initial release surge is likely due to quercetin molecules adsorbed on or proximate to the niosomal surface, which diffuse swiftly into the surrounding medium owing to their superficial positioning. Such an immediate release phase could prove beneficial in clinical applications, potentially facilitating a more rapid onset of therapeutic action.

**FIGURE 4:**
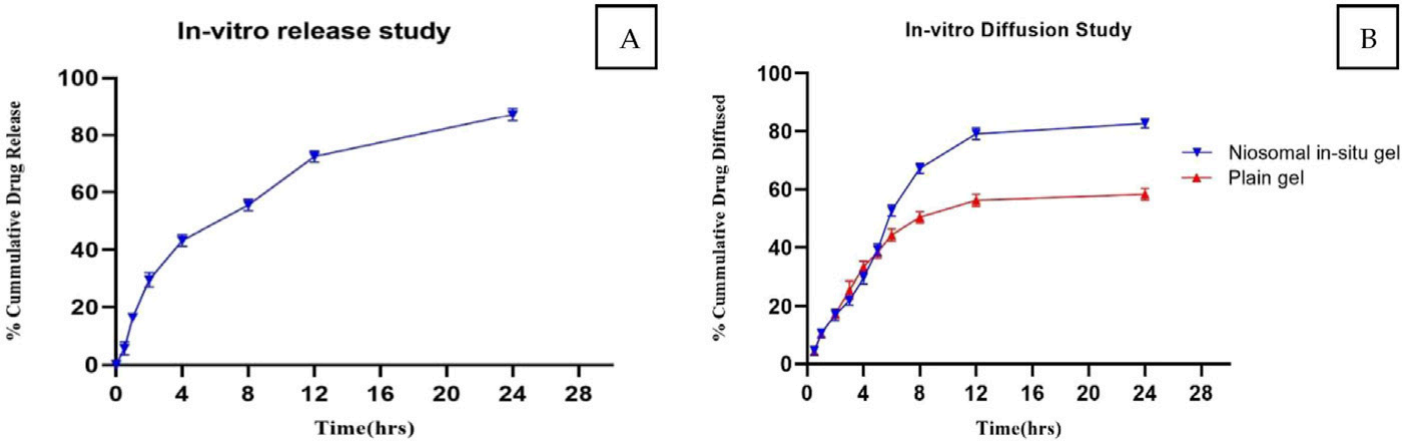
**(A)** *In Vitro* Release Profile of Quercetin-Loaded Niosomes for the Optimized Batch. **(B)** Comparative diffusion study of niosomal *in situ* gel and plain gel.

Subsequently, the release profile transitioned into a sustained release phase, characterized by a gradual release of quercetin, culminating in an overall cumulative release of 87.16% by the end of the 24-hour period, as presented in Figure 4. This prolonged release phase is attributed to the gradual diffusion of quercetin encapsulated within the lipid bilayers, which mediates a controlled release and may extend therapeutic effects over an extended period.

To elucidate the underlying kinetics of quercetin release from the niosomal structure, various mathematical models were employed. Curve-fitting analyses revealed that the release process predominantly adhered to first-order kinetics, demonstrated by a strong regression coefficient (*R* ^2^ = 0.968), indicating that the release rate is directly proportional to the concentration of quercetin within the formulation.

Further analysis of the release data using the Higuchi model—a model that describes release as a diffusion-controlled mechanism from a homogenous matrix—indicated a linear fit, as shown in Table 6 and corresponding figure, suggesting that the quercetin release was diffusion-driven. The alignment of the release profile with both first-order and Higuchi kinetics supports a mechanism whereby an initial rapid release is succeeded by a sustained, diffusion-driven phase, aligning with the anticipated properties of niosomal drug delivery systems.

**TABLE 6:** Kinetic analysis of niosomal formulations.

Kinetic model	*R* ^2^
Zero order	0.9158
First order	0.9164
Higuchi	0.982
Kors-peppas	0.7705
Hixson	0.9071

The observed biphasic release pattern, encompassing an initial rapid phase followed by extended release, highlights the potential of quercetin-loaded niosomes to offer a prompt therapeutic dose, succeeded by continuous release. This characteristic is particularly advantageous in enhancing quercetin’s bioavailability and therapeutic efficacy in the treatment of Parkinson’s disease. These findings underscore the promise of this formulation as a feasible intranasal drug delivery approach, advancing neuroprotective treatment modalities. Additionally, the release pattern conformed to the Higuchi model as shown in Table 6 and Figure (Supplementary File) (Syed et al., 2021; El-Diasty et al., 2020; Hong et al., 2020).

### 3.7 Stability study

The stability of the optimized quercetin-loaded niosomal formulation was meticulously evaluated over a 3-month period to assess its robustness and shelf-life under standard storage conditions. The formulation was stored at room temperature (25°C ± 2°C) and monitored at regular intervals to determine any variations in key characteristics, namely particle size, entrapment efficiency, and polydispersity index (PDI) as shown in Table 7. These parameters are critical indicators of the physical stability of the niosomal system, as fluctuations in size, drug retention, or homogeneity could impact the formulation’s efficacy, bioavailability, and suitability for therapeutic use.

**TABLE 7:** Characterization of optimized niosomal dispersion at 0 Months and after 3 Months of storage at room temperature.

	Vesicle size (nm)	% Entrapment efficiency	Zeta potential (mV)	% Drug release	Polydispersity index
0 months	187	79.08	−31.08	0.223	86.7
3 months	210	76.2	−29.3	0.25	82.3

Particle Size: Particle size stability is crucial, as it directly influences the delivery and release profile of the formulation. Consistency in particle size indicates that the niosomes maintain structural integrity without aggregation or degradation of vesicles over time. Throughout the 3-month storage period, particle size measurements were conducted at defined intervals to observe any potential increase due to vesicle fusion or particle aggregation.

Entrapment Efficiency (EE): Entrapment efficiency is a measure of the percentage of quercetin encapsulated within the niosomal vesicles relative to the initial amount. Stability in EE over time indicates that the formulation retains quercetin effectively without significant leakage or degradation.

### 3.8 Evaluation of *in situ* gel

The flowability of the formulation, represented by the gelation time required for phase transition from sol to gel, was recorded in Table 8. The optimized *in situ* gel formulation demonstrated a drug content of 94% w/v, while its pH was slightly alkaline at 6.8, as shown in Table 9. Determining the gelation temperature, a critical step in the development of thermoreversible *in situ* gels, revealed that using 19% w/v Poloxamer 407 yielded optimal results with a transition temperature of 32°C–33°C; this temperature varied with polymer concentrations between 17% and 23% (Syed et al., 2021; El-Diasty et al., 2020). The optimized niosomal *in situ* gel exhibited excellent rheological properties, with a sol viscosity of 57.52 cP and a gel viscosity of 1937.33 cP (Syed et al., 2021), as detailed in Table 9. *In vitro* diffusion studies compared the quercetin release profiles of a quercetin-loaded niosomal *in situ* gel and a plain gel over 24 h. The plain gel, with unencapsulated quercetin, displayed a slower diffusion profile, achieving only a 58.27% cumulative release, limited by its lack of niosomal encapsulation. Conversely, the niosomal *in situ* gel demonstrated a biphasic release pattern, with an initial burst release of 39.23% in the first 5 h, followed by sustained release, reaching 82.74% by 24 h. This enhanced permeation is due to niosomes encapsulating quercetin within lipid bilayers, which increase solubility and facilitate membrane interaction, allowing for controlled, prolonged drug release, which is beneficial for applications requiring consistent bioavailability.

**TABLE 8:** Determination of flowability through gelation time.

Concentration of poloxamer 407	Time required in seconds
17%	No gelation
19%	45
21%	58
23%	63

**TABLE 9:** pH, Gelation temperature, Viscosity for sol and gel value of the *in situ* gel.

Trails	pH	Gelation temperature	Viscosity for sol (Cps)	Viscosity for gel (Cps)
1	6.8	34	42.08	1980
2	6.5	31.5	50.7	1937
3	6.6	33.5	79.8	1895
Average	6.63	34.13	57.52	1937.33
SD	±0.21	±0.21	16.13	34.7

Mean ± SD (N = 3).

### 3.9 *In Vivo* characterization

#### 3.9.1 Behavioural parameter

**FIGURE 5:**
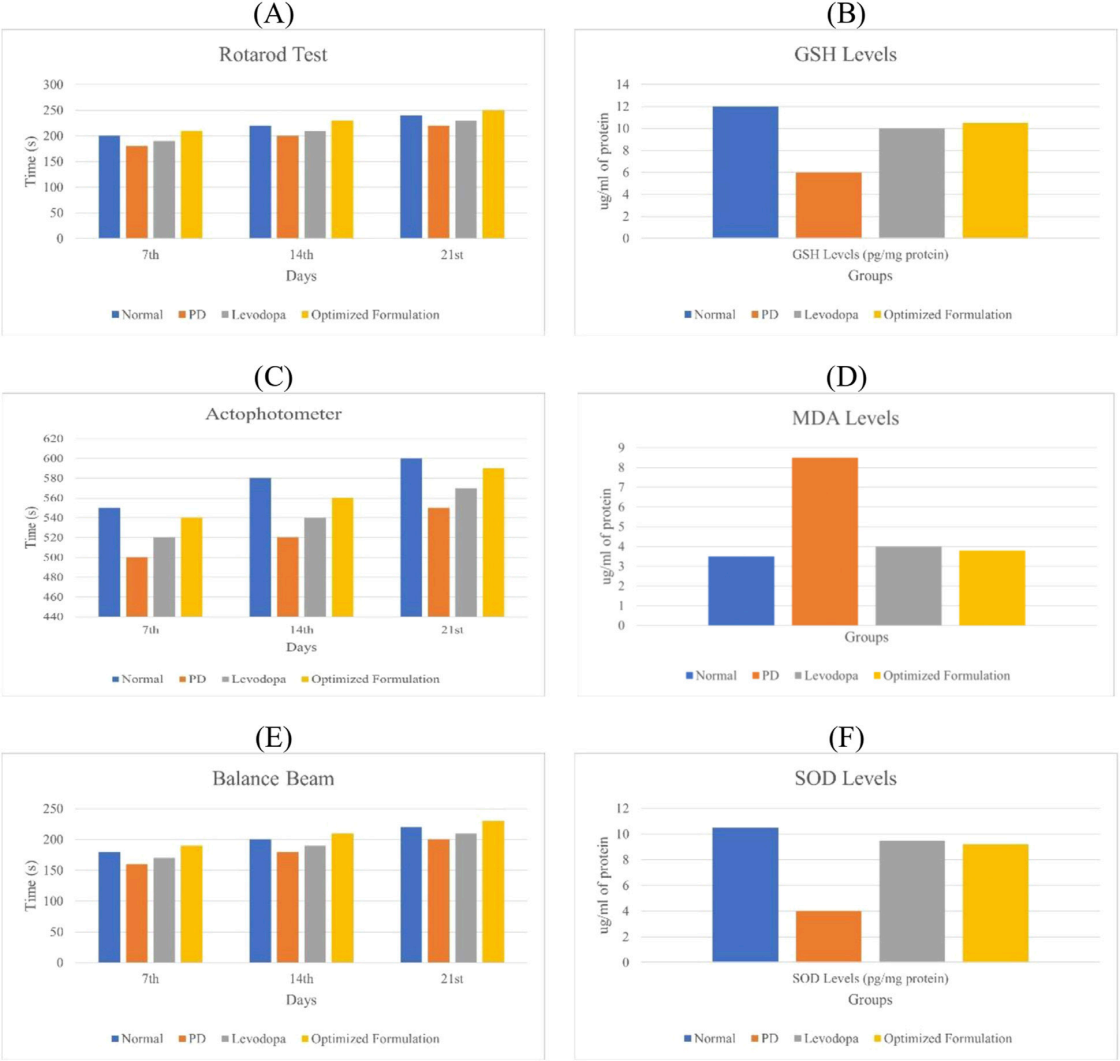
**(A)** Rotarod performance of different animal groups. **(C)** Effect of quercetin-loaded niosomes on actophotometer scores in rats. **(E)** Effect of quercetin-loaded niosomes on balance beam performance in rats. **(B)** Glutathione (GSH) estimation in the rat brain. **(D)** Malondialdehyde (MDA) estimation in the rat brain. **(F)** Superoxide dismutase (SOD) estimation in the rat brain.

Behavioural parameters like rotarod, actophotometer, and balance beam are assessed in Parkinson’s disease to evaluate motor coordination, activity levels, and balance, which are commonly impaired in the condition and the scores of each parameter is depicted in Figure 5 (Hong et al., 2020).

#### 3.9.2 Rotarod analysis

On day 0, no significant differences in rotarod performance were observed across the groups, establishing a baseline for motor coordination. By day 14, however, the disease group exhibited a substantial decline in performance compared to the normal group, reflecting the motor impairments characteristic of Parkinson’s disease. In contrast, both the standard treatment and experimental treatment groups showed marked improvements in performance relative to the disease group, indicating that the treatments effectively enhance motor coordination and balance, potentially mitigating Parkinsonian motor deficits. By day 21, the disease group continued to demonstrate significantly impaired performance, highlighting persistent motor dysfunction. Both treatment groups maintained their enhanced performance, underscoring the therapeutic potential of these interventions in alleviating Parkinson’s symptoms, particularly bradykinesia (Jamwal et al., 2017).

#### 3.9.3 Actophotometer analysis

On day 7, there were no significant differences in actophotometer activity counts across the groups, indicating comparable baseline activity levels. By day 14, the disease group showed a significant reduction in activity counts compared to the normal group, which aligns with the motor deficits observed in Parkinson’s disease. Both the standard and treatment groups exhibited significant improvements in activity counts, reflecting positive effects on motor activity and coordination. On day 21, the disease group continued to show significantly lower activity counts compared to the normal group, reaffirming the persistence of Parkinson’s symptoms in the absence of intervention. However, both treatment groups maintained elevated activity counts, reflecting sustained therapeutic benefits in improving motor dysfunctions, as shown in Figure 5 (Jaiswal et al., 2016).

#### 3.9.4 Balance beam test

On day 7, there were no significant differences in balance beam traversal times across the groups, indicating no early variations in motor performance. By day 14, the disease group exhibited a significant increase in traversal time compared to the normal group, suggesting the balance and coordination deficits typical of Parkinson’s pathology. Both the standard and treatment groups showed significantly reduced traversal times relative to the disease group, indicating improvements in balance and motor function. By day 21, the disease group continued to demonstrate prolonged traversal times, while both treatment groups maintained improved traversal times, demonstrating the efficacy of the treatments in alleviating Parkinson’s disease symptoms, particularly issues related to balance and coordination (Jamwal et al., 2017).

#### 3.9.5 Estimation of glutathione (GSH)

The disease group exhibited a significant decrease in GSH levels compared to the normal group. Both the standard and treatment groups showed elevated GSH levels, indicating a neuroprotective effect. The increase in GSH levels was highly significant for both the standard and treatment groups when compared to the disease group, as illustrated in Figure 5A (Jaiswal et al., 2016).

#### 3.9.6 Estimation of malondialdehyde (MDA)

The disease group displayed significantly elevated levels of MDA compared to the normal group. In contrast, both the standard and treatment groups exhibited reduced MDA levels compared to the disease group, indicating a neuroprotective effect. The reduction in MDA levels was significant in both the standard and treatment groups when compared to the disease group, as shown in Figure 5B (Jaiswal et al., 2016).

#### 3.9.7 Estimation of superoxide dismutase (SOD)

The disease group demonstrated significantly reduced SOD levels compared to the normal group. Both the standard and treatment groups exhibited increased SOD levels relative to the disease group, suggesting a neuroprotective effect. The increase in SOD levels was significant for both the standard and treatment groups compared to the disease group, as depicted in Figure 5C (Jaiswal et al., 2016).

### 3.10 Statistical analysis

The *p*-values for all the behavioral and biochemical assay results were less than 0.001, highlighting significant differences between the disease group and the treatment groups. These results underscore the efficacy of the treatments in mitigating Parkinson’s disease-related impairments and neurodegeneration.

### 3.11 Histopathology of hippocampus of rat brain

The examination of a section of the cerebral cortex stained with hematoxylin and eosin (H&E) reveals several critical observations: (A) Cortical neurons (↑) characterized by rounded vesicular nuclei (yellow ↑) display sharply demarcated nuclear contours, while blood vessels (v) are accompanied by a narrow perivascular space indicative of degenerated and shrunken neurons. Notably, the disease group exhibits a significantly elevated histological score in comparison to the normal group, reflecting an increase in cortical neurons with rounded vesicular nuclei and neuropil exhibiting sharply demarcated nuclei or signs of meningeal involvement (Syed et al., 2021; Jaiswal et al., 2016).

**FIGURE 6:**
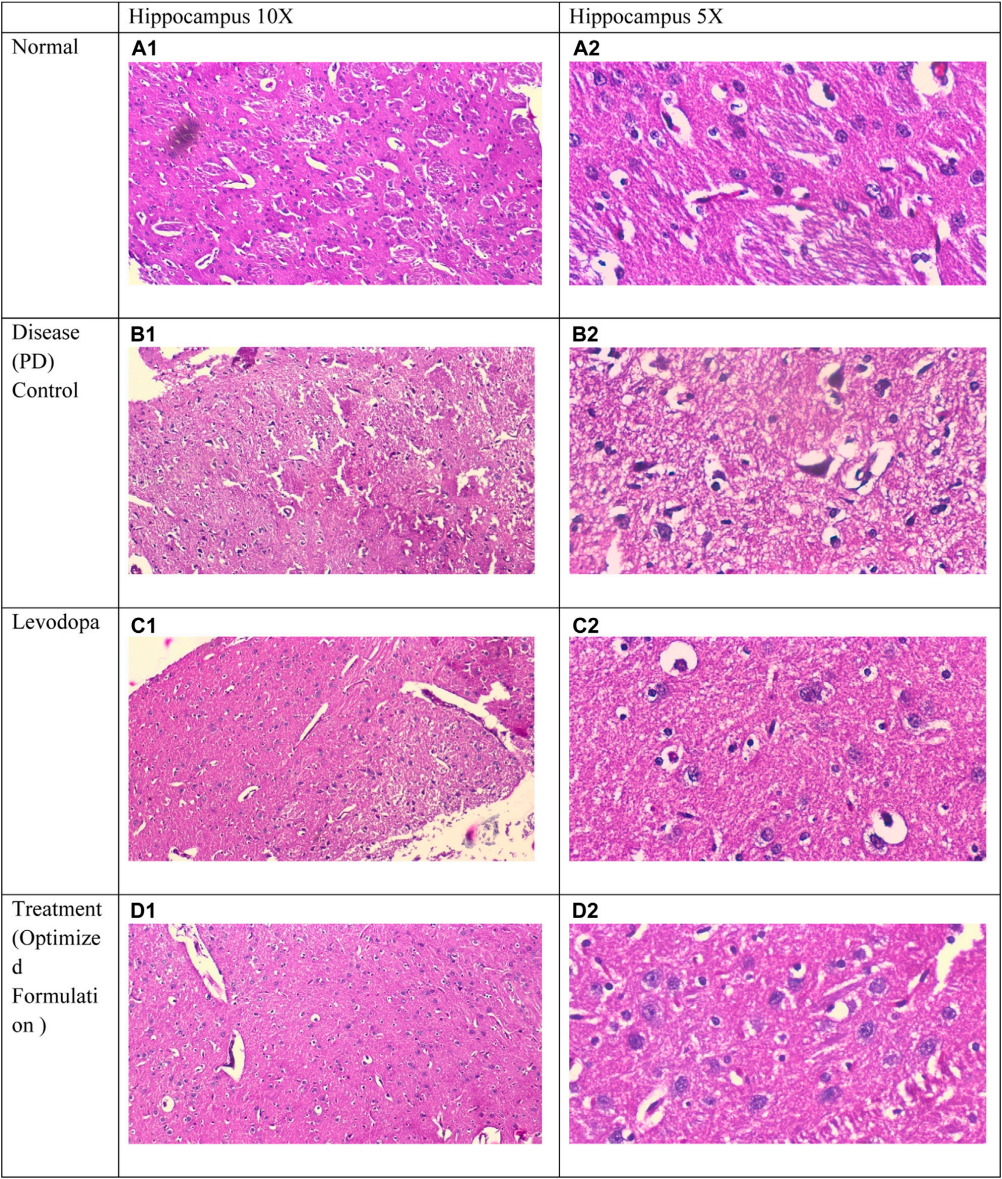
Comparative Histological Analysis of Cerebral Tissue Across Study Groups : **(A1, A2)** depict normal parenchyma with intact morphology and no pathological changes. **(B1, B2)** indicate severe gliosis, edema, neuronal loss, and vessel congestion in the positive control group. **(C1, C2)** display reduced gliosis and edema in the standard group. **(D1, D2)** from the treatment group demonstrate further improvement, with minimal gliosis and no vascular congestion.

Figure 6 (A1) and (A2) depict the normal parenchyma morphology, featuring oval-shaped cells with prominent nucleoli and pale basophilic cytoplasm, devoid of significant gliosis, edema, neuronal loss, or congestion of meningeal vessels. In stark contrast, Figure 6 (B1) and (B2), representative of the positive control group, reveal cerebral tissue marked by pronounced gliosis, edema, and congestion of meningeal vessels, along with observable neuronal loss and occasional neutrophilic infiltration. Figure 6 (C2) and (C3), corresponding to the standard group, illustrate a slight reduction in both gliosis and edema, with an absence of neutrophils or meningeal vessel congestion. Likewise, Figure 6 (D1) and (D2), belonging to the treatment 1 group, demonstrate diminished gliosis and edema, also lacking neutrophils and meningeal vessel congestion (Hong et al., 2020; Jamwal et al., 2017; Jaiswal et al., 2016).

## 4 Discussion

Parkinson’s disease (PD) stands as the second most prevalent neurodegenerative disorder, predominantly characterized by motor symptoms such as tremors, bradykinesia, postural instability, and rigidity. These motor manifestations are often preceded by a range of non-motor symptoms, including sleep disturbances, olfactory deficits, cognitive impairments, and depression, which can significantly impact patients’ quality of life (Madiha et al., 2021). Quercetin, a polyphenolic bioflavonoid found abundantly in fruits and vegetables, possesses powerful antioxidant and anti-inflammatory properties, presenting a promising neuroprotective candidate for PD treatment (Wrobel-Biedrawa et al., 2022; Grewal et al., 2021). Quercetin effectively neutralizes reactive oxygen species (ROS), enhances endogenous antioxidant defences, and modulates inflammatory pathways, which are crucial in slowing neurodegeneration (Wrobel-Biedrawa et al., 2022). Additionally, it has demonstrated efficacy in inhibiting α-synuclein aggregation, a pathological hallmark of PD, further underscoring its potential role in neuroprotection (Grewal et al., 2021).

However, quercetin’s therapeutic application is challenged by its classification as a Biopharmaceutical Classification System (BCS) Class IV compound, signifying poor solubility and permeability, which limits its bioavailability and clinical effectiveness (Sirvi et al., 2022). To overcome these limitations, innovative formulation strategies, such as niosomal encapsulation, offer a viable approach by enhancing quercetin’s solubility and permeability, thereby improving its bioefficacy in PD models (Madiha et al., 2021; Nirmal et al., 2010). In this study, we developed an intranasal *in situ* gel formulation of quercetin aimed at overcoming these delivery challenges. This formulation capitalizes on the intranasal route, which enables direct brain targeting and improved bioavailability, potentially offering more effective therapeutic outcomes for PD patients by ensuring efficient central nervous system drug delivery.

The formulation of niosomes was achieved through the ethanol injection method, incorporating a fixed amount of Quercetin along with varied ratios of Span (Sp) and cholesterol (CH). Using Design Expert^®^ software (version 13, Stat-Ease Inc., Minneapolis, MN, United States), a three-factor, three-level (3³) Box-Behnken Design (BBD). Increased cholesterol concentration competes with the drug for bilayer space, resulting in a larger hydrodynamic diameter due to a more structured bilayer (Shah et al., 2020). Cholesterol stabilizes bilayers by ordering lipid chains, thus enhancing encapsulation efficiency for hydrophilic drugs and decreasing drug release rates by reinforcing membrane stability (Uchegbu and Vyas, 1998). Conversely, increased Span 60 concentration and sonication time effectively reduced particle size. Span 60, with its long alkyl chain (C18), lowers surface tension, aiding in smaller vesicle formation and providing bilayer rigidity for more stable particles (Essa, 2010; Owodeha-Ashaka et al., 2010). The ultrasound waves applied during sonication break larger vesicles into smaller, uniform ones through cavitation (Khallaf et al., 2020). This combination of Span 60 and sonication yielded optimized niosomes with suitable particle size for brain delivery (Abdelkader et al., 2014; Shah et al., 2020; Nasr et al., 2020).

Cholesterol concentration was also positively correlated with encapsulation efficiency (EE%), due to its impact on enhancing the mechanical strength, cohesion, and permeability of lipid bilayers in niosomes. Elevated cholesterol levels reduce vesicle fluidity, thus imparting rigidity and lowering permeability—an advantage under high-stress conditions (Moghassemi and Hadjizadeh, 2014). Conversely, an increase in Span 60 and sonication adversely influenced EE, likely due to hydrogen bonding interactions between cholesterol and Span 60 within the bilayer, restricting drug encapsulation volume despite reduced vesicle size (Moghassemi and Hadjizadeh, 2014). Span 60, characterized by its long C18 chain and low water solubility, promotes tight packing in the bilayer, but this also limits the drug volume encapsulated (Masjedi and Montahaei, 2021). Cholesterol helps seal potential leakage pathways within bilayer membranes, creating a more robust structure as surfactants densely pack during formulation (Junyaprasert et al., 2008; Zolghadri et al., 2023).

The type of surfactant, charged species presence, and storage conditions notably impact niosome zeta potential (Durán-Lobato et al., 2022). With an increase in Span 60, zeta potential magnitude decreases, attributable to the surfactant’s hydrophobic nature, which weakens electrostatic repulsion and encourages aggregation (Shah et al., 2012). While sonication time has less impact on zeta potential, further studies are warranted to clarify its effects. Images of the Quercetin-loaded niosomes showed a spherical morphology with a uniform size distribution, signifying niosomal stability, well-defined structure, and rigid bilayer. Stability is driven by hydrogen bonding interactions between Span 60 ester groups and cholesterol hydroxyl groups (Syed et al., 2021; El-Diasty et al., 2020). The formulation, stored at room temperature (25°C ± 2°C), was regularly evaluated for particle size, entrapment efficiency, and polydispersity index (PDI) to ensure physical stability, as these parameters affect therapeutic efficacy, bioavailability, and formulation suitability.

Particle size stability is essential as it affects the formulation’s delivery and release profile. Consistent particle size indicates preserved vesicle integrity without fusion or aggregation over time. Entrapment efficiency (EE%) stability suggests effective retention of quercetin without significant leakage, with all evaluations indicating a sustained capacity for drug encapsulation and a robust, physically stable formulation.

Behavioral assessments, including the rotarod, actophotometer, and balance beam tests, are pivotal tools for evaluating motor coordination, activity, and balance deficits in Parkinson’s disease models, as these impairments are central features of the condition. The rotarod test, in particular, serves as a robust indicator of motor dysfunction, often revealing characteristic symptoms such as balance disturbances and motor incoordination in untreated Parkinson’s models. Such impairments align with the hallmark symptoms of bradykinesia and muscle rigidity that arise from dopaminergic neurodegeneration. Enhanced rotarod performance in groups receiving either standard or experimental treatments suggests that these interventions may alleviate motor symptoms, thereby reinforcing their clinical potential in the therapeutic management of Parkinson’s disease (Hong et al., 2020; Jamwal et al., 2017).

The actophotometer test complements this analysis by measuring overall activity levels, which often decline in Parkinson’s disease, reflecting motor inactivity and fatigue typical of the condition. Lower activity counts in untreated groups highlight the motor impairment intrinsic to Parkinson’s pathology, mirroring the apathy and reduced physical engagement observed clinically. Conversely, elevated activity levels in treatment groups suggest that these interventions may effectively restore normal motor activity, thus underscoring their importance in managing the disease’s debilitating motor symptoms (Jaiswal et al., 2016).

The balance beam test further elucidates motor coordination deficits, with traversal time serving as a metric for postural and balance impairments. In untreated models, prolonged traversal times reflect significant coordination difficulties, comparable to the postural instability seen in Parkinson’s patients, where such balance issues substantially impact patient safety and quality of life. Improved traversal times in treatment groups indicate that these therapies may help mitigate balance issues, reducing fall risk and improving patient outcomes (Jamwal et al., 2017).

Oxidative stress, a critical component of Parkinson’s pathology, was evaluated through biochemical assays of markers such as glutathione (GSH), malondialdehyde (MDA), and superoxide dismutase (SOD). Lower levels of GSH and SOD in untreated groups correlate with weakened antioxidant defenses typical of neurodegenerative conditions. An increase in these antioxidant markers in treatment groups points to a neuroprotective effect, suggesting that these interventions bolster antioxidant capacity. Conversely, elevated MDA levels in untreated models signal increased lipid peroxidation, a sign of oxidative damage. Treatments that reduce MDA levels may offer neuroprotection by lessening oxidative stress, a factor crucial for slowing disease progression (Jaiswal et al., 2016).

Histopathological analysis of the hippocampus corroborates neurodegeneration in Parkinson’s disease models, with untreated groups displaying markers such as gliosis, edema, and cortical neuron degeneration. These pathological changes reflect inflammatory processes and neuronal loss characteristic of Parkinsonian neurodegeneration. Reduced gliosis and edema in treated groups suggest that neuroprotective agents may counteract neuroinflammatory processes, thereby mitigating cellular damage in affected brain regions. The observed absence of gliosis and inflammation in these groups highlights the potential of these therapies to curb pathological changes central to Parkinson’s progression (Syed et al., 2021; Jaiswal et al., 2016; Hong et al., 2020).

Parkinson’s disease (PD), marked by motor and non-motor symptoms, severely impacts patients’ quality of life. Quercetin, with antioxidant and anti-inflammatory properties, shows neuroprotective potential for PD by mitigating oxidative stress and inhibiting α-synuclein aggregation. Its therapeutic application benefits from intranasal delivery using niosomes, which enhance bioavailability and stability, improving quercetin’s efficacy in PD models.

## 5 Conclusion

In summary, the study successfully formulated and characterized quercetin-loaded niosomes with desirable properties such as optimal particle size, high entrapment efficiency, and stable zeta potential. The *in vitro* and *in vivo* results indicate that these niosomes can significantly enhance the bioavailability and therapeutic efficacy of quercetin. The study confirms that quercetin-loaded niosomes are a promising delivery system for neuroprotection in Parkinson’s disease. Future research should focus on further optimizing the formulation for large-scale production and exploring the detailed mechanisms underlying the observed pharmacodynamic effects. Additionally, long-term stability studies and clinical trials are necessary to validate the efficacy and safety of quercetin-loaded niosomes in human subjects.

## Data Availability

The original contributions presented in the study are included in the article/supplementary material, further inquiries can be directed to the corresponding author.
